# Effects of Substitution, and Adding of Carbohydrate and Fat to Whey-Protein on Energy Intake, Appetite, Gastric Emptying, Glucose, Insulin, Ghrelin, CCK and GLP-1 in Healthy Older Men—A Randomized Controlled Trial

**DOI:** 10.3390/nu10020113

**Published:** 2018-01-23

**Authors:** Caroline Giezenaar, Yonta van der Burgh, Kylie Lange, Seva Hatzinikolas, Trygve Hausken, Karen L. Jones, Michael Horowitz, Ian Chapman, Stijn Soenen

**Affiliations:** 1 Discipline of Medicine and National Health and Medical Research Council of Australia (NHMRC) Centre of Research Excellence in Translating Nutritional Science to Good Health, Adelaide Medical School, Adelaide, SA 5000, Australia; caroline.giezenaar@adelaide.edu.au (C.G.); yonta.vanderburgh@wur.nl (Y.v.d.B.); kylie.lange@adelaide.edu.au (K.L.); seva.hatzinikolas@adelaide.edu.au (S.H.); karen.jones@adelaide.edu.au (K.L.J.); michael.horowitz@adelaide.edu.au (M.H.); ian.chapman@adelaide.edu.au (I.C.); 2 Department of Medicine, Haukeland University Hospital, 5021 Bergen, Norway; trygve.hausken@helse-bergen.no

**Keywords:** whey protein, energy intake, gastric emptying, gut hormones, aging

## Abstract

Protein-rich supplements are used widely for the management of malnutrition in the elderly. We reported previously that the suppression of energy intake by whey protein is less in older than younger adults. The aim was to determine the effects of substitution, and adding of carbohydrate and fat to whey protein, on ad libitum energy intake from a buffet meal (180–210 min), gastric emptying (3D-ultrasonography), plasma gut hormone concentrations (0–180 min) and appetite (visual analogue scales), in healthy older men. In a randomized, double-blind order, 13 older men (75 ± 2 years) ingested drinks (~450 mL) containing: (i) 70 g whey protein (280 kcal; ‘P_280_’); (ii) 14 g protein, 28 g carbohydrate, 12.4 g fat (280 kcal; ‘M_280_’); (iii) 70 g protein, 28 g carbohydrate, 12.4 g fat (504 kcal; ‘M_504_’); or (iv) control (~2 kcal). The caloric drinks, compared to a control, did not suppress appetite or energy intake; there was an increase in total energy intake (drink + meal, *p* < 0.05), which was increased most by the M_504_-drink. P_280_- and M_504_-drink ingestion were associated with slower a gastric-emptying time (*n* = 9), lower ghrelin, and higher cholecystokinin (CCK) and glucagon-like peptide-1 (GLP-1) than M_280_ (*p* < 0.05). Glucose and insulin were increased most by the mixed-macronutrient drinks (*p* < 0.05). In conclusion, energy intake was not suppressed, compared to a control, and particularly whey protein, affected gastric emptying and gut hormone responses.

## 1. Introduction

Over recent decades, the prevalence of malnutrition, both under-nutrition and obesity, has increased in older men and women in Western societies [1,2]. A growing awareness of the prevalence and adverse effects of the major muscle loss that occurs during aging, irrespective of body mass index (BMI, kg/m^2^)—e.g., reduced functional capacity and decreased quality of life [1,3,4]—has led to the development of nutritional strategies designed specifically to preserve and/or restore skeletal muscle mass and function. A ‘common’ strategy is the use of supplements, which are usually high-energy drinks rich in whey protein [5,6,7,8,9].

Despite this increasing use of protein-rich drinks, information about their effects on energy intake and underlying gastrointestinal mechanisms in older people is limited. In younger adults, preloads high in protein suppress appetite and energy intake [10,11,12,13,14,15] more than iso-caloric preloads high in fat or carbohydrate. In young adults, variations in gut hormone secretion and/or action (e.g., ghrelin, cholecystokinin (CCK) and glucagon-like polypeptide-1 (GLP-1)), as well as gastric emptying, are likely to regulate energy intake [16,17,18,19,20,21,22].

Compared to younger adults, healthy older people exhibit decreased taste and food palatability, are less hungry and fuller during the fasting and postprandial states, and consume less food and energy [23]. This has been termed the ‘anorexia of aging’ [3,4]. Healthy aging is also associated with reduced responsiveness to the suppressive effects of nutrients on appetite and energy intake [24,25,26,27]. We have recently demonstrated that acute administration of 30 g (120 kcal), and 70 g (280 kcal) whey protein drinks, 180 min before a meal, suppressed subsequent energy intake by 12–17% in young, but without suppression in healthy older, men [24] so that in older men protein ingestion increased total energy intake (drink plus energy intake) compared to a control (~0 kcal) to a greater extent than in the young men. Gastric emptying of the whey protein drink was shown to be slower in older than younger men [24]. In young adults, gastric emptying of 500 mL of protein (375 kcal) has been reported to be comparable to carbohydrate (400 kcal) or fat (375 kcal), when expressed as the rate of emptying in mL/min [28], as well as slower when ingested as a mixed macronutrient dairy breakfast (high-protein compared to high-carbohydrate; ~400 kcal) [29]. The high-protein dairy breakfast had higher plasma CCK and GLP-1 responses—gut hormones known to slow gastric emptying [29].

The aim of this study was to determine the effects of substitution and addition of carbohydrate and fat to whey protein on ad libitum energy intake at a buffet meal, gastric emptying, gut hormones and perceptions of appetite and gastrointestinal symptoms, in healthy older men. We hypothesized that the replacement of protein by carbohydrate and fat would result in less suppression of subsequent energy intake, more rapid gastric emptying, more pronounced changes in plasma gut hormone concentrations (insulin, ghrelin, CCK, GLP-1), and that the addition of carbohydrate and fat would result in greater suppression of subsequent energy intake, slower gastric emptying, more pronounced changes in plasma gut hormone concentrations and decreased perceptions of appetite, compared to a ‘pure’ whey—protein drink.

## 2. Materials and Methods

Thirteen older healthy men, 65 years or older (mean ± standard error of the mean (SEM); age: 75 ± 2 years; body weight: 79 ± 2 kg; height: 1.75 ± 0.01 m; BMI: 26 ± 1 kg/m^2^), were recruited by advertisement. Subjects were excluded if they failed to comprehend the study protocol, had donated blood in the 12 weeks prior to the study days, had known lactose intolerance or food allergies, or were undernourished (score < 24 on the Mini Nutritional Assessment [30]). Further exclusion criteria were low plasma ferritin levels, diabetes, gallbladder or pancreatic disease, significant gastrointestinal symptoms (abdominal pain, gastro-esophageal reflux, diarrhea, or constipation) or surgery, depression (score ≥ 11 on the Geriatric Depression Questionnaire [31]), alcohol abuse, smoking, use of illicit substances or medications known to potentially affect energy intake, or had impaired cognitive function (score < 25 on Mini Mental State [32]). The Royal Adelaide Hospital Human Research Ethics Committee approved the study protocol and the study was conducted in accordance with the Declaration of Helsinki. The study is a sub-analysis of a larger study, which is registered as a clinical trial with the Australian New Zealand Clinical Trial Registry (www.anzctr.org.au; ACTRN12614000846628). All subjects provided written informed consent prior to their inclusion.

### 2.1. Protocol

Subjects were studied on 4 occasions, separated by 3–14 days, to determine the effects of drinks (~450 mL) containing either: (i) 70 g whey protein (280 kcal; ‘P_280_’); (ii) 14 g whey protein, 28 g carbohydrate, 12.4 g fat (280 kcal; ‘M_280_’); (iii) 70 g protein, 28 g carbohydrate, 12.4 g fat (504 kcal; ‘M_504_’); or (iv) an iso-palatable control drink (~2 kcal; ‘control’) on energy intake, gastric emptying, gut hormones, and perceptions of appetite and gastrointestinal symptoms, in a randomized (using the method of randomly permuted blocks; www.randomization.com), double-blind, cross-over design.

Drinks were prepared on the morning of the study day, by homogenizing olive oil (Bertolli Australia Pty Ltd., Unilever Australasia, Sydney, NSW, Australia) and dissolving whey protein isolate (Fonterra Co-Operative Group Ltd., Palmerston North, New Zealand) and dextrose, in varying volumes of demineralized water and diet lime cordial (Bickford’s Australia Pty Ltd., Salisbury South, SA, Australia), to achieve the desired composition, by a research officer (SH) who was not involved in the data analysis. Both the investigator and the subject were blinded to the treatment. The drinks were matched for taste and served in a covered cup.

Before each study day, subjects consumed a standardized meal (beef lasagne (McCain Foods Pty Ltd., Wendouree, VIC, Australia), containing ~591 kcal), at ~19:00 p.m. Thereafter, subjects fasted overnight from solids and liquids until they attended the laboratory at ~08:30 a.m. Subjects refrained from strenuous physical activity for 24 h before the study day.

Subjects removed all metal objects and were seated in an upright position on a wooden chair. An intravenous cannula was inserted for blood sampling and subsequent measurement of glucose and gut hormones. In each subject, blood samples and ultrasound measurements of gastric volume, and perceptions of appetite and gastrointestinal symptoms were performed before (during fasting) and after ingestion of the drink, until 180 min. Subjects were instructed to consume the drink within 2 min. At 180 min, each subject was presented with a standard, cold, buffet-style meal, in excess of what they were expected to consume (total energy content of 2.457 kcal; 19% protein, 50% carbohydrates, 31% fat), in a room by themselves to limit external distractions, and were allowed to eat for 30 min (180–210 min) until comfortably full.

### 2.2. Measurements

#### 2.2.1. Energy Intake

The buffet-style meal consisted of bread, chicken, ham, cheese, margarine, mayonnaise, yoghurt, custard, fruit, fruit salad, orange juice, iced coffee and water [27]. The amount eaten at the meal (g) was quantified by weighing the food before and after consumption. Energy intake (kcal), as intake at the buffet meal, and as the cumulative energy intake, defined as the sum of energy intake at the buffet meal and the energy content of the preload drink, proportions of protein, carbohydrate and fat (Foodworks version 8; Xyris Software Pty Ltd., Spring Hill, QLD, Australia), and change in energy intake at the buffet meal (expressed as % of energy intake of the control day) by a given protein load, compared to the control, were calculated.

#### 2.2.2. Gastric Emptying by 3D Ultrasonography

Total gastric volume was measured by a Logiq™ 9 ultrasound system (GE Healthcare Technologies, Sydney, NSW, Australia) with TruScan Architecture (built-in magnetically-sensored 3D positioning and orientation measurement (POM)), including a 3D sensor, attached to a 3.5C broad spectrum 2.5–4 MHz convex transducer, and a transmitter, placed at the level of the stomach, immediately behind the subject, at 0, 5, 15, 30, 45, 60, 75, 90, 105, 120, 135, 150, 165 and 180 min [33]. The stomach was scanned along its longitudinal axis, whilst the subject was holding their breath and sitting still. Stomach volumes were calculated using EchoPAC-3Dsoftware (GE Vingmed Sound, Horten, Norway). Intragastric retentions were calculated as total gastric volume minus fasting gastric volume (baseline) at each time point, and expressed as percentage of the maximal gastric volume (100%). Data were imput by linear interpolation when ultrasound images lacked sufficient clarity [27]. The rate of gastric emptying was calculated during each 15-min interval in the early phase (i.e., 0–60 min), and the late phase (i.e., 60 min until 100% emptying time per individual). Fifty percent of the gastric emptying time (T50; min) and ‘complete’ (residual volume of the drink in the stomach was ≤5%) gastric emptying time (100% gastric emptying time; T100; min) were calculated [27].

#### 2.2.3. Blood Glucose and Plasma Insulin, Ghrelin, Cholecystokinin (CCK) and Glucagon-Like Peptide-1 (GLP-1) Concentrations

Blood samples were collected, at 0, 5, 15, 30, 45, 60, 90, 120, 150, 180 min, into ice-chilled, EDTA-coated tubes. No inhibitors were added [33]. Plasma was obtained by centrifugation for 15 min, at 3200 rpm, at 4 °C, and samples were stored at −80 °C for further analysis of hormone concentrations.

Blood glucose (millimoles per liter) was determined immediately after collection, by the glucose oxidase method, using a portable glucometer (Optium Xceed, Abbott Laboratories, Sydney, NSW, Australia). Intra- and inter-assay coefficients of variation were 2.6% and 15.2%.

Total plasma insulin (milliunits per liter) was measured by enzyme-linked immunosorbent assay (ELISA) immunoassay (10-1113; Mercodia, Uppsala, Sweden). The minimum detectable limit was 1.0 mU/L. Intra- and inter-assay coefficients of variation were 3.0% and 6.8%.

Total plasma ghrelin (picograms per milliliter) was measured using a radioimmunoassay (RIA) with some modifications to a published method [34]. The radiolabel was supplied by Perkin Elmer (NEX388, Boston, MA, USA). The standard and samples were incubated with the antibody and radiolabel for 3–4 days, at 4 °C. The detection limit was 40 pg/mL. Intra- and inter-assay coefficients of variation were 5.1% and 10.1%.

Plasma CCK-8 (picomoles per liter) was measured by RIA, using an adaption of a previous method [35]. Samples were extracted in 66% ethanol; extracts were dried down and re-suspended in assay buffer (50 mM phosphate, 10 mM EDTA, 2 g/L gelatin, pH = 7.4). Standards were prepared using synthetic sulphated CCK-8 (Sigma Chemical, St Louis, MO, USA), antibody (C2581, Lot 105H4852, Sigma Chemical) was added at a working dilution of 1/17,500 and sulphated CCK-8 ^125^I-labeled with Bolton and Hunter reagent (Perkin Elmer, Boston, MA, USA) was used as tracer. Incubation was for 7 days at 4 °C. The antibody bound fraction was separated by the addition of dextran-coated charcoal containing gelatin (0.015 g gelatin, 0.09 g dextran, 0.15 g charcoal in 30 mL assay buffer), and the radioactivity was determined in the supernatants following centrifugation. The detection limit was 1 pmol/L. The intra- and inter-assay coefficients of variation were 8.1% and 11.5%.

Total plasma GLP-1 (picomoles per liter) was measured by RIA (GLPIT-36HK; Millipore, Billerica, MA, USA), with a detection limit of 3 pmol/L. Intra - and inter -assay coefficients of variation were 2.7% and 7.1%.

Peak/nadir and time to peak/nadir concentrations for glucose, insulin, ghrelin, CCK and GLP-1 were calculated for the caloric drink conditions.

#### 2.2.4. Perceptions of Appetite and Gastrointestinal Symptoms

Perceptions of hunger, desire to eat, prospective consumption, fullness, nausea and bloating were rated using a visual analogue scale (VAS) questionnaire, at 0, 5, 15, 30, 45, 60, 75, 90, 105, 120, 135, 150, 165, 180 and 210 min [36]. The questionnaire consisted of 100-mm horizontal lines, where 0 represented that the sensation was ‘not felt at all’ and 100 represented that the sensation was ‘felt the greatest’. Subjects placed a vertical mark on each horizontal line to indicate the strength of each sensation at the specified time points.

### 2.3. Data and Statistical Analyses

On the basis of our previous work, with an observed within-subject standard deviation (SD) of 267 kcal for suppression of energy intake by whey protein, and 31 min for gastric emptying half time [24], we calculated that 13 subjects would allow detection of a within-group difference between treatments for suppression in energy intake of 272 kcal and T50 of 35 min, with power equal to 0.8 and alpha equal to 0.05.

Statistical analyses were performed using SPSS software (version 22; IBM, Armonk, NY, USA). Differences between study conditions for energy intake, gastric emptying, perceptions of appetite and gastrointestinal symptoms (visual analogue scores) and glucose and hormone concentrations were determined using one-way repeated-measures ANOVA, with the treatment as the within-subject factor. Post hoc comparisons were adjusted with the Bonferroni method. Interaction effects of time by treatment, for concentrations of blood glucose and plasma insulin, ghrelin, CCK and GLP-1, and perceptions of hunger, desire to eat, prospective food consumption, fullness, nausea and bloating, were determined using a two-way repeated measures ANOVA, with treatment and time as the within-subject factors. Within-subject correlations were determined using a general linear model with fixed slope and random intercept [37]. Areas under the curve (AUC) for gastric emptying, perceptions of appetite and gastrointestinal symptoms, and concentrations of glucose, insulin, ghrelin, CCK and GLP-1, were calculated from baseline to 60 min (i.e., ‘early’ phase of gastric emptying) and 60 to 180 min (i.e., ‘late’ phase of gastric emptying), using the trapezoidal rule. Peak/nadir and time to peak/nadir perceptions of hunger, desire to eat, prospective food consumption, fullness, nausea and bloating were calculated for the all conditions. Assumptions of normality were verified for all outcomes before the statistical analysis. Statistical significance was accepted at *p* < 0.05. All data are presented as mean values ± SEMs.

## 3. Results

The study protocol was well-tolerated by all subjects. Baseline gastric volumes (mean ± SEM of four study days: 33 ± 4 mL), blood glucose (5.7 ± 0.1 mmol/L), plasma insulin (5.1 ± 1.8 mU/L), ghrelin (1659 ± 165 pg/mL), CCK (2.0 ± 0.2 pmol/L) and GLP-1 concentrations (15 ± 1 pmol/L), and perceptions of hunger (31 ± 13 mm), desire to eat (30 ± 12 mm), prospective food consumption (46 ± 14 mm), fullness (2 ± 1 mm), nausea (3 ± 1 mm) and bloating (3 ± 1 mm), were not different between study days.

### 3.1. Energy Intake

**Figure 1:**
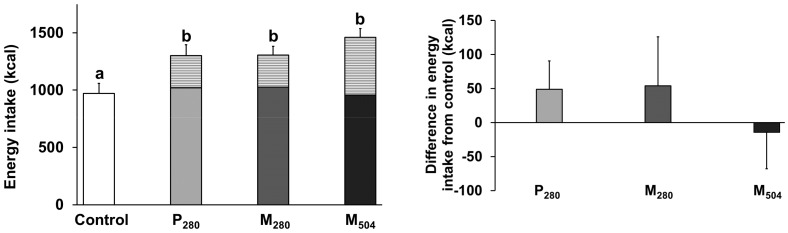
**Left**: mean (±standard error of the mean (SEM)) energy intake at the buffet meal (kcal) in healthy older men (*n* = 13) after drinks (~450 mL; energy content of the drink as the striped part of each bar) containing either: (i) 70 g whey protein (280 kcal; ‘P_280_’); (ii) 14 g protein, 28 g carbohydrate, 12.4 g fat (280 kcal; ‘M_280_’); (iii) 70 g protein, 28 g carbohydrate, 12.4 g fat (504 kcal; ‘M_504_’); or (iv) an iso-palatable control drink (~2 kcal; ‘control’). **Right**: mean (±SEM) suppression of energy intake after caloric drinks (P_280_, M_280_ and M_504_) compared to control. ^a,b^ *p* < 0.05 Total energy intakes (meal plus drink) for P_280_, M_280_ and M_504_ (^b^) were higher compared to the control (^a^).

Ad libitum energy intake at the buffet meal (Figure 1) and energy percentages of protein, carbohydrate and fat were not different between study days (mean of four study days: energy intake: 994 ± 76 kcal, *p* = 0.53; protein: 20 ± 0.4%, *p* = 0.60; carbohydrate: 52 ± 1%, *p* = 0.25; fat: 30 ± 1% *p* = 0.83). There was no suppression of energy intake by the caloric drinks, compared to the control (*p* > 0.05). Total energy intake (drink plus meal) was higher after all caloric drinks, compared to the control (*p* < 0.001; post hoc tests versus control (972 ± 87 kcal): P_280_ (1300 ± 95kcal) *p* < 0.001; M_280_ (1306 ± 76 kcal) *p* = 0.003; M_504_ (1461 ± 76 kcal) *p* < 0.001).

### 3.2. Gastric Emptying

**Figure 2:**
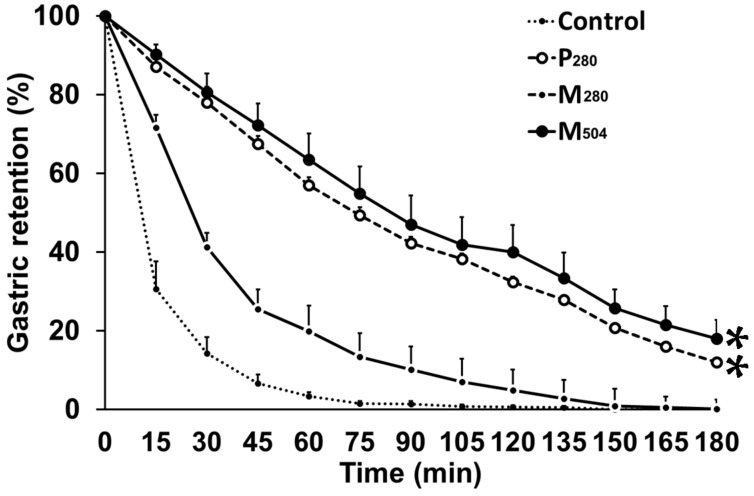
Mean (±SEM) gastric retention (%) in healthy older men (*n* = 9), after drinks containing either: (i) 70 g whey protein (280 kcal; ‘P_280_’; dashed line with open circles); (ii) 14 g protein, 28 g carbohydrate, 12.4 g fat (280 kcal; ‘M_280_’; solid line with open circles); (iii) 70 g protein, 28 g carbohydrate, 12.4 g fat (504 kcal; ‘M_504_’; solid line with closed circles); or (iv) an iso-palatable control drink (~2 kcal; ‘control’; dotted line). Gastric emptying half time (T50) was higher after P_280_ and M_504_, compared to M_280_ and control (* *p* < 0.05).

In four subjects, the quality of ultrasound stomach images was insufficient to determine gastric emptying in one or more conditions, and all data related to gastric emptying in these subjects were, therefore, excluded from the analysis. The control and the M_280_ drink emptied in an overall non-linear pattern, whereas the pattern of emptying of P_280_ and M_504_ was linear (Figure 2). Gastric emptying of P_280_ and M_504_ was slower *p* < 0.001), and gastric retention greater (*p* < 0.001), than M_280_, with 50% gastric emptying times being ~three-fold higher (*p* < 0.001, Table 1).

### 3.3. Blood Glucose and Plasma Gut Hormone Concentrations

#### 3.3.1. Glucose

**Figure 3:**
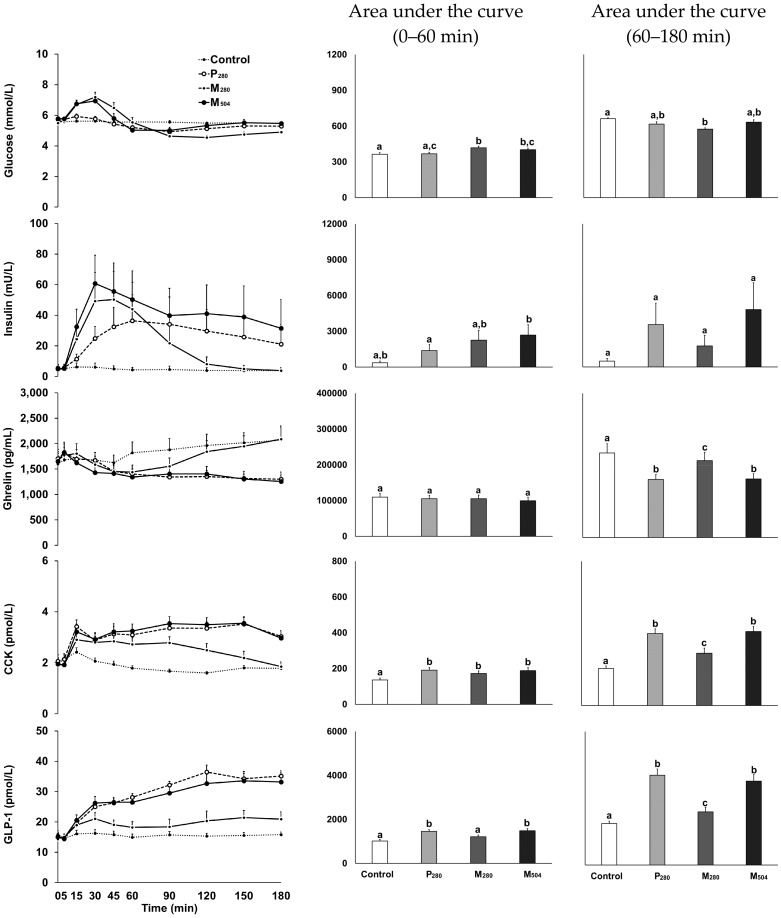
Mean (±SEM) concentrations of blood glucose and plasma insulin, ghrelin, cholecystokinin (CCK) and glucagon-like peptide-1 (GLP-1) in healthy older men (*n* = 13), after drinks containing either: (i) 70 g whey protein (280 kcal; ‘P_280_’; dashed line with open circles); (ii) 14 g protein, 28 g carbohydrate, 12.4 g fat (280 kcal; ‘M_280_’; solid line with open circles); (iii) 70 g protein, 28 g carbohydrate, 12.4 g fat (504 kcal; ‘M_504_’; solid line with closed circles); or (iv) an iso-palatable control drink (~2 kcal; ‘control’; dotted line). There was an interaction effect of time by drink condition for concentrations of blood glucose (*p* < 0.001), insulin (*p* = 0.038), ghrelin (*p* < 0.001), CCK (*p* < 0.001) and GLP-1 (*p* < 0.001). Different letters indicates significant difference (*p* < 0.05) in area under the curves (0–60 or 60–180 min) between drink-conditions: control vs. P_280_ vs. M_280_ vs. M_504_.

Blood glucose concentrations increased after M_280_ and M_504_, returned to baseline ~60 min, and stayed below baseline until the buffet meal (interaction effect of time by drink condition: *p* < 0.001). Peak glucose concentrations were higher after M_280_ and M_504_, compared to P_280_ (*p* = 0.001, Supplementary Table S1). Early phase AUC_0–60 min_ glucose concentrations were higher after M_280_ and M_504_, compared to the control, and after M_280_, compared to P_280_ (*p* < 0.001, Figure 3). Late phase AUC_60–180 min_ glucose concentrations were lower after M_280_ than the control (drink condition effect: *p* < 0.001). Glucose concentrations at 180 min were lower after M_280_ compared to control and M_504_ (*p* = 0.003).

#### 3.3.2. Insulin

Plasma insulin concentrations increased after all caloric drinks (P_280_, M_280_ and M_504_; interaction effect of time by drink condition: *p* = 0.038). The mixed-macronutrient drinks evoked a rapid increase in insulin, insulin peak concentrations were higher after M_280_ and M_504_ compared to P_280_ (*p* < 0.001, Supplementary Table S1), and the drinks containing 70 g of whey protein (P_280_ and M_504_) remained elevated (Figure 3). Early phase AUC_0–60 min_ insulin concentrations were higher after M_504_, compared to P_280_ (*p* = 0.008).

#### 3.3.3. Ghrelin

Plasma ghrelin concentrations decreased after all caloric drinks (P_280_, M_280_ and M_504_; interaction effect of time by drink condition: *p* < 0.001). Nadir ghrelin concentrations were lower after P_280_ and M_504_, compared to M_280_, (*p* = 0.001, Supplementary Table S1), and remained suppressed after P_280_ and M_504_ (Figure 3). Late phase AUC_60–180 min_ ghrelin concentrations were lower after all caloric drinks, compared to the control, and after P_280_ and M_504_, compared to M_280_ (*p* < 0.001). Ghrelin concentrations at 180 min were lower after M_504_ and P_280_, compared to the control and M_280_ (*p* < 0.001).

#### 3.3.4. CCK

Plasma CCK concentrations increased after all caloric drinks (P_280_, M_280_ and M_504_; interaction effect of time by drink condition: *p* < 0.001). Peak CCK concentrations were higher after P_280_ and M_504_, compared to M_280_ (*p* < 0.001, Supplementary Table S1), and remained elevated after P_280_ and M_504_ (Figure 3). Early phase AUC_0–60 min_ CCK concentrations were higher after the caloric drinks, compared to the control (*p* < 0.001). Late phase AUC_60–180 min_ CCK concentrations were higher after the caloric drinks, compared to the control, and after P_280_ and M_504_, compared to M_280_ (*p* < 0.001). CCK concentrations at 180 min were higher after P_280_ and M_504_, compared to the control and M_280_ (*p* < 0.001).

#### 3.3.5. GLP-1

Plasma GLP-1 concentrations increased after all caloric drinks (P_280_, M_280_ and M_504_; interaction effect of time by drink condition: *p* < 0.001, Figure 3). Early phase AUC_0–60 min_ GLP-1 concentrations were higher after P_280_ and M_504_, compared to the control and M_280_ (*p* < 0.001). Late phase AUC_60–180 min_ GLP-1 concentrations were higher after all caloric drinks, compared to the control, and after P_280_ and M_504_, compared to M_280_ (*p* < 0.001). GLP-1 concentrations at 180 min were higher after the caloric drinks, compared to the control, and after P_280_ and M_504_, compared to M_280_ (*p* < 0.001).

### 3.4. Correlations between Gastric Retention and Hormones

Plasma insulin (AUC 0–180 min; *r* = 0.41 *p* = 0.029) ghrelin (*r* = −0.70 *p* < 0.001), CCK (*r* = 0.76 *p* < 0.001) and GLP-1 (*r* = 0.78 *p* < 0.001) concentrations were, within subjects, related to gastric emptying (AUC 0–180 min).

### 3.5. Perceptions of Appetite and Gastrointestinal Symptoms

Early phase AUC_0–60 min_ and late phase AUC_60–180 min_ perceptions of hunger, desire to eat, prospective food consumption, fullness, nausea, and bloating were not different between study days (*p* > 0.05, Supplementary Figure S1). Hunger (mean decrease over four study visits: 9 ± 2 mm, time to nadir: 29 ± 7 min, time effect: *p* < 0.001), desire to eat (9 ± 1 mm, 21 ± 5 min, *p* < 0.001) and prospective food consumption (11 ± 2 mm, 35 ± 7 min, *p* < 0.001) initially decreased after drink ingestion and increased thereafter to ratings higher than baseline, immediately before the buffet meal (180 min). Fullness (mean increase over four study days: 16 ± 5 mm, time to peak: 38 ± 8 min, *p* = 0.001) increased after the drink to return to baseline thereafter. Nausea and bloating did not change over time (nausea: *p* = 0.51, bloating: *p* = 0.10).

## 4. Discussion

This study examined the effects of substituting fat and protein for, or adding them to, whey protein, on energy intake, gastric emptying, blood glucose and plasma gut hormone concentrations, perceptions of appetite and gastrointestinal symptoms in healthy older men. The major novel observation is that ingestion of a whey protein drink of 280 kcal (P_280_: 70 g protein) or a mixed macronutrient drink of 504 kcal (M_504_: 70 g protein, 28 g carbohydrate, 12.4 g fat,) was associated with slower gastric emptying, lower ghrelin, and higher CCK and GLP-1 concentrations than an iso-caloric mixed-macronutrient drink (M_280_: 14 g protein, 28 g carbohydrate, 12.4 g fat: 280 kcal). There was no suppression of energy intake or appetite by the caloric drinks, compared to the control.

The use of high protein supplements by older people is widespread, and increasing, in response to greater awareness of the prevalence of undernutrition and sarcopenia in older people [38] and evidence that protein supplementation may increase muscle mass and function [39,40]. If timing and preparation are optimized, it may be possible to give sufficient protein (probably at least 35 g [41]) to older people, to preserve, or increase muscle mass and function, without suppressing energy intake. Indeed, our observations suggest that optimal protein administration may increase overall energy intake in older people. None of the caloric drinks suppressed subsequent ad libitum energy intake at a buffet meal, compared to a non-caloric control, and consequently, there was an increase in total energy intake. This observation is consistent with our recent finding that the suppression of subsequent energy intake by oral ingestion and intraduodenal infusions of whey protein is less in healthy older men (~1%) than in young controls (~15–19%) [24,25]. Total energy intake (drink plus meal) was predictably increased most by the drink with the highest energy content (504 kcal)—a substantial increase of ~50% or 490 kcal, compared with an increase of ~34% or ~330 kcal after both 280 kcal drinks. Comparable amounts of protein could reasonably be given as protein supplements several times during the day. We have reported that variation in the timing of protein ingestion does not affect energy intake at a subsequent meal in healthy older people, and that total energy intake is higher on the protein days compared to a control [42]. These findings raise the intriguing possibility that appropriately designed protein supplements, administered in divided doses, might increase energy intake in undernourished people by meaningful amounts (>300–500 kcal/day), without the need to encourage and supervise additional energy intake.

We, and others, have shown that healthy aging is associated with modest slowing of gastric emptying of both solids and liquids, although the rate of emptying generally remains within the relatively wide normal range for young subjects (i.e., ~1–4 kcal/min) [24,43,44,45]. In healthy older men, the addition of 28 g carbohydrate and 12.4 g fat to the 70 g (280 kcal) whey protein did not affect gastric emptying time; both P_280_ and M_504_ had comparable T50 and T100 and therefore, the rate of gastric emptying was higher for M_504_ than P_280_ (e.g., initial rates of gastric emptying of ~3 and 2 kcal/min, respectively). Iso-caloric substitution of 56 g (224 kcal) protein with carbohydrate and fat resulted in faster gastric emptying; M_280_ compared to P_280_ had lower T50 and T100 and thus a faster rate of gastric emptying (~4 vs. 2 kcal/min).

The M_280_ drink had emptied completely, and P_280_ and M_504_ were ~90% emptied immediately before the meal. It should be appreciated that, as the subjects were seated, it is possible that, despite being mixed for ~45 min prior to, until immediately before, consumption, the fat (olive oil) separated from the protein/carbohydrate solution, and emptied from the stomach slower than the aqueous phase, by ‘layering’ on the denser aqueous components [46].

The hormones, insulin, ghrelin, GLP-1 and PYY, are secreted by the gastrointestinal tract, in response to the ingested nutrients. Plasma gut hormone concentrations were, within subjects, related to gastric retention; lower ghrelin, and higher insulin, CCK and GLP-1 concentrations correlated with slower gastric emptying. Both drinks containing 70 g protein (P_280_ and M_504_) had comparable gut hormone responses, which were greater than the responses evoked by the M_504_ drink. These observations suggest that in healthy older men, gastric emptying and plasma gut hormone concentrations were more likely dependent on the amount of protein, rather than the energy content of the drink.

In young subjects, the addition of protein to a glucose meal increases the insulin response [47,48,49]. It has been reported that whey protein, which has a high content of insulinotropic amino acids [50], resulted, when compared to casein protein, in a higher increase of plasma insulin concentrations [51]. In our study, both mixed-macronutrient drinks evoked a rapid increase in plasma insulin concentrations, while insulin remained elevated for longer in the drinks containing 70 g of whey protein. Glucose concentrations immediately before the meal were lower after M_280_ than M_504_, which is likely related to the M_504_ drink still being emptied from the stomach.

In young people, it has been reported previously that effects were larger and more sustained after high compared to low protein on ghrelin concentrations [29,52,53,54,55], protein compared to glucose [29,53], but not fat [56], on CCK concentrations, and protein compared to carbohydrate [29,57,58,59] or fat [57,59,60]—in several studies but not all studies [61,62,63]—on GLP-1 concentrations. In our study, the drinks containing 70 g of protein resulted in a comparably more sustained lower decrease in ghrelin concentrations and higher increases in CCK and GLP-1 than M_280_, which is likely to be related to the potency of the higher content of whey protein, and for the M_504_ drink, an additional caloric content.

Our study has several limitations. The subject numbers were relatively small. This applies particularly to the gastric emptying measurements (*n* = 9). Nevertheless, the findings were clear-cut. We studied only men, as they appear to have the greatest ability to regulate energy intake in response to energy manipulation [26], and in women, particularly the menstrual cycle may have a confounding effect on appetite and energy intake. We have also recently reported that there is no effect of gender on gastric emptying, concentrations of glucose or gut hormones, perceptions of appetite and gastrointestinal symptoms in older people [64]. Energy intake at the buffet meal was assessed three hours after drink ingestion, to allow for complete emptying of the drinks from the stomach, and not during the remainder of the day—accordingly, potential compensatory changes in energy intake after lunch were not evaluated. While the drinks were palatable and matched for taste, we did not assess the subjects’ perceptions of taste and/or pleasantness of the drinks. Blood glucose was measured by a glucometer, which is less than optimal; however, the results appeared clear-cut.

## 5. Conclusions

A drink containing 70 g whey-protein (280 kcal), and a mixed-macronutrient drink containing 70 g protein, 28 g carbohydrate and 12.4 g fat (504 kcal), were associated with slower gastric emptying time, lower ghrelin, and higher CCK and GLP-1 concentrations than a mixed-macronutrient drink containing 14 g protein, 28 g carbohydrate and 12.4 g fat (280 kcal). The caloric drinks did not suppress energy intake, compared to the non-caloric control and, consequently, there was an increase in total energy intake, particularly with the mixed-macronutrient drink with the highest caloric content. Our findings are likely to have implications for the composition of protein-rich supplements, for both undernourished and obese older people as well as for targeting gastric emptying and gut hormone responses by preload intakes, in relation to, for example, glycemic control in older people.

## Figures and Tables

**Table 1:** Gastric emptying parameters after drink ingestion in healthy older men.

Gastric emptying parameters	Control	P_280_	M_280_	M_504_
50% emptying time (T50; min)	12 ± 2 ^a^	78 ± 11 ^b^	26 ± 2 ^a^	93 ± 13 ^b^
100% emptying time (T100; min)	58 ± 7 ^a^	180 ± 0 ^b^	120 ± 8 ^c^	170 ± 7 ^b^
Gastric retention (%)				
Area under curve (AUC)_0–60 min_	1546 ± 200 ^a^	4666 ± 222 ^b^	2979 ± 83 ^c^	4871 ± 240 ^b^
AUC_60–180 min_	94 ± 42 ^a^	3907 ± 559 ^b^	552 ± 122 ^c^	4331 ± 771 ^b^
Rate of gastric emptying (kcal/min) ^1^				
Early phase		2.0 ± 0.3 ^a^	3.7 ± 0.1 ^b^	3.1 ± 0.6 ^b^
Late phase		1.1 ± 0.1^a^	1.3 ± 0.3 ^a^	2.2 ± 0.3 ^b^
Amount emptied (%)				
at 60 min	98 ± 1 ^a^	61 ± 11 ^b^	86 ± 4 ^a^	56 ± 12 ^b^
at 180 min	100 ± 0 ^a^	89 ± 3 ^b^	100 ± 0 ^a^	85 ± 5 ^b^

Mean (±SEM) 50% and 100% emptying time (min), gastric retention (%), rate of gastric emptying (kcal/min) and amount emptied (%) at 60 and 180 min in healthy older men (*n* = 9), after drinks containing either: (i) 70 g whey protein (280 kcal; ‘P_280_’); (ii) 14 g protein, 28 g carbohydrate, 12.4 g fat (280 kcal; ‘M_280_’); (iii) 70 g protein, 28 g carbohydrate, 12.4 g fat (504 kcal; ‘M_504_’); or (iv) an iso-palatable control drink (~2 kcal; ‘control’). ^1^ The rate of gastric emptying was calculated as the mean of rates of emptying during each 15-min interval, respectively, in the early phase (i.e., 0–60 min) and late phase (i.e., 60 min until 100% emptying time per individual). Different letters indicate a significant difference (*p* < 0.05) between drink conditions; gastric emptying time and retention and amount emptied were higher after P_280_ and M_504_ (^b^) than M_280_ (^a,c^) and control (^a^), rate of gastric emptying was higher after M_504_ (^b^) and M_280_ (^a,b^) than P_280_ (^a^).

## Supplementary Materials

The following are available online at http://www.mdpi.com/2072-6643/10/2/113/s1, Figure S1: Hunger, desire to eat, prospective food consumption, fullness, nausea and bloating after drink ingestion in healthy older men, Table S1: Glucose, insulin, ghrelin, CCK and GLP-1 after drink ingestion in healthy older men.
