# Supplementary material for: Effects of Substitution, and Adding of Carbohydrate and Fat to Whey-Protein on Energy Intake, Appetite, Gastric Emptying, Glucose, Insulin, Ghrelin, CCK and GLP-1 in Healthy Older Men—A Randomized Controlled Trial
Source: Nutrients. 2018 Jan 23;10(2):113. doi: 10.3390/nu10020113 (PMC5852689; doi:10.3390/nu10020113)
Supplement: Supplementary file 1 [file nutrients-10-00113-s001.pdf]

Supplementary Table S1. Glucose, insulin, ghrelin, CCK and GLP-1 after drink ingestion in healthy older men.

Glucose and gut hormones	Control	P_{280}	M_{280}	M_{504}
Peak/nadir concentration				
Glucose	5.9 ± 0.1 [a]	6.1 ± 0.1 [a,b]	7.4 ± 0.3 [b]	7.2 ± 0. 3 [b]
Insulin	7 ± 3 [a]	39 ± 17 [a,b]	56 ± 18 [a,b]	67 ± 18 [b]
Ghrelin	2171 ± 240 [a]	1855 ± 183 [a]	2194 ± 251[b]	1862 ± 198 [b]
CCK	2.7 ± 0.2 [a]	3.9 ± 0.3 [b]	3.1 ± 0.3 [a,b]	3.9 ± 0.3 [b]
GLP-1	19 ± 1 [a]	39 ± 3 [b]	25 ± 2 [a]	36 ± 3 [b]
Time to peak/nadir				
Glucose	42 ± 13	20 ± 5	29 ± 3	22 ± 2
Insulin	15 ± 3 [a]	53 ± 7 [b]	39 ± 3 [b]	44 ± 7 [b]
Ghrelin	44 ± 13 [a]	126 ± 14 [b]	58 ± 7 [a]	114 ±18 [b]
CCK	39 ±15 [a]	87 ± 16 [a,b]	45 ± 12 [a,b]	99 ± 16 [b]
GLP-1	87 ± 22 [a]	138 ± 13 [a,b]	98 ± 18 [a,b]	155 ± 7 [b]
180-min concentration				
Glucose	5.5 ± 0.1 [a]	5.3 ± 0.4 [a]	4.9 ± 0.4 [b]	5.5 ± 0.4 [a]
Insulin	3.9 ± 1.9	21 ± 13	3.8 ± 1.6	32 ± 9
Ghrelin	2069 ± 257 [a]	1302 ± 137 [a,b]	2091 ± 259 [a]	1254 ± 117 [b]
CCK	1.8 ± 0.1 [a]	3.0 ± 0.2 [b]	1.9 ± 0.2 [a]	3.0 ± 0.3 [b]
GLP-1	16 ± 1 [a]	35 ± 2 [b]	21 ± 2 [a]	33 ± 3 [b]

Mean (± SEM) peak/nadir concentrations, time to peak/nadir (min) and 180-min concentrations of blood glucose and plasma insulin, ghrelin, CCK and GLP-1 in healthy older men (n = 13), after drinks containing either: (i) 70 g whey protein (280 kcal; 'P_{280}'), (ii) 14 g protein, 28g carbohydrate, 12.4 g fat (280 kcal; 'M_{280}'), (iii) 70 g protein, 28 g carbohydrate, 12.4 g fat (504 kcal; 'M_{504}'), or (iv) an iso-palatable control drink (~2 kcal; 'control'). [a,b,c,d] $p < 0.05$, post hoc test: different letter indicates significant difference between drink-conditions: control vs. P_{280} vs. M_{280} vs. M_{504}.

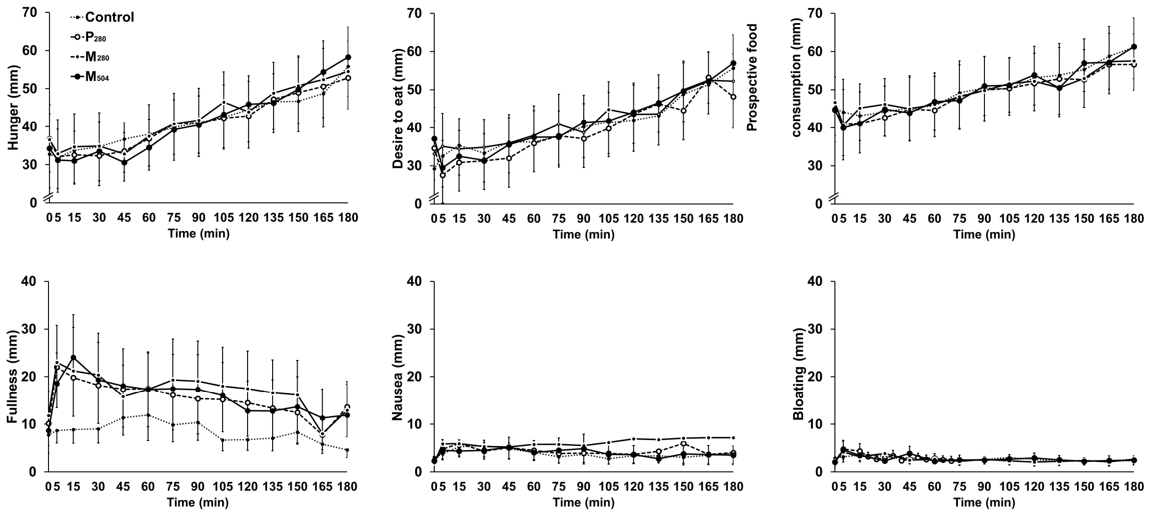

Supplementary Figure S1. Mean (± SEM) visual analogue score (VAS, mm) of hunger, desire to eat, prospective food consumption, fullness, nausea and bloating in healthy older men (n = 13), after drinks containing either: (i) 70 g whey protein (280 kcal; 'P_{280}'; dashed line with open circles), (ii) 14 g protein, 28 g carbohydrate, 12.4 g fat (280 kcal; 'M_{280}'; solid line with open circles), (iii) 70 g protein, 28 g carbohydrate, 12.4 g fat (504 kcal; 'M_{504}'; solid line with closed circles), or (iv) an iso-palatable control drink (~2 kcal; 'control'; dotted line).

Effects of time were significant for hunger ($p < 0.001$), desire to eat ($p < 0.001$), prospective food consumption ($p < 0.001$) and fullness ($p = 0.001$).
